# Potentiation of Natural Killer Cells for Cancer Immunotherapy: A Review of Literature

**DOI:** 10.3389/fimmu.2017.01061

**Published:** 2017-09-01

**Authors:** Lacy E. Lowry, William A. Zehring

**Affiliations:** ^1^Department of Basic Science, Geisinger Commonwealth School of Medicine, Scranton, PA, United States; ^2^Department of Biochemistry, Geisinger Commonwealth School of Medicine, Scranton, PA, United States

**Keywords:** cancer, immunology, immunotherapy, natural killer cells, cellular heterogeneity

## Abstract

It is widely acknowledged that the human immune system plays a crucial role in preventing the formation and progression of innumerable types of cancer (1). The mechanisms by which this occurs are numerous, including contributions from both the innate and adaptive immune systems. As such, immunotherapy has long been believed to be an auspicious solution in the treatment of malignancy (2). Recent research has highlighted the promise of natural killer (NK) cells as a more directed immunotherapy approach. This paper will focus on the methods of potentiation of NK cells for their use in cancer therapy.

## Introduction

From the beginning, cancer treatment approaches with chemotherapy and radiation therapy have largely been unable to discriminate between healthy and malignant cells in their killing action. These therapeutics therefore often sacrifice healthy tissue in the effort to treat the cancer, which too often results in temporary, but often harsh side effects, and long-term consequences, including secondary malignancy. In addition, these treatment methods fail to account for cellular heterogeneity between individual patients with the same diagnosis, or even within a single tumor itself. Although still controversial, evidence indicates that in certain cancer types, small portions of cancer cells situated within an individual tumor display “stem-cell” like properties and are resistant to such therapy, such that they continue to proliferate in spite of treatment (3).

Cancer immunotherapy seeks to strengthen and direct the patient’s natural immune mechanisms against malignant cells, with the aim of targeting the disease while minimizing effects to surrounding healthy tissue (1). Preliminary data display the potential of immunotherapy, specifically natural killer (NK) cell-based immunotherapy, for targeting the quiescent cancer stem cell (CSC) population (3). Although such approaches have been discussed for decades, only recent advances in our understanding of cancer immunology have allowed for direct applications regarding these more specialized therapies (2).

## What is in a Tumor: That Which I Call Heterogeneity

“Tumor heterogeneity” refers to differences between tumors of the same type in different patients, as well as to differences among cancer cells within the same tumor. Both can lead to diverse responses to therapy and may explain why some tumor cells remain following aggressive treatment (4). There are two predominant theories to explain tumor heterogeneity, the CSC, or hierarchical model, alluded to above, and the stochastic model. The CSC model argues that individual tumors are comprised of a combination of both tumorigenic and non-tumorigenic cells, which are derived from a single CSC that undergoes transformation as a consequence of both genetic and epigenetic influences. Theory holds that the vast majority of the cells in these cancers have little capacity to contribute to disease progression, thus it is necessary to focus therapy on the small subpopulations of tumorigenic cells rather than the tumor as a whole (5). By contrast, in the stochastic model, all tumor cells are considered biologically equivalent but vary in behavior and function based upon intrinsic and extrinsic influences (6). Although there is compelling evidence to support the CSC theory in many types of cancer, including leukemias, breast cancers, brain cancers, and colon cancers, debate continues as to whether this model can be applied widely to all cancers, or whether certain cancer types exhibit the stochastic model (5).

## Muster Your Wits and Stand in Your Own Defense: The Immune System

The human immune system is divided into two arms, innate and adaptive, which work cohesively in response to specific internal and external stimuli. The innate immune system is involved in immediate host defense to perceived pathogens and includes neutrophils, monocytes, macrophages, complement, cytokines, and acute phase proteins. Adaptive immunity consists of antigen-specific reactions mediated by T lymphocytes and B lymphocytes.

**Figure 1:**
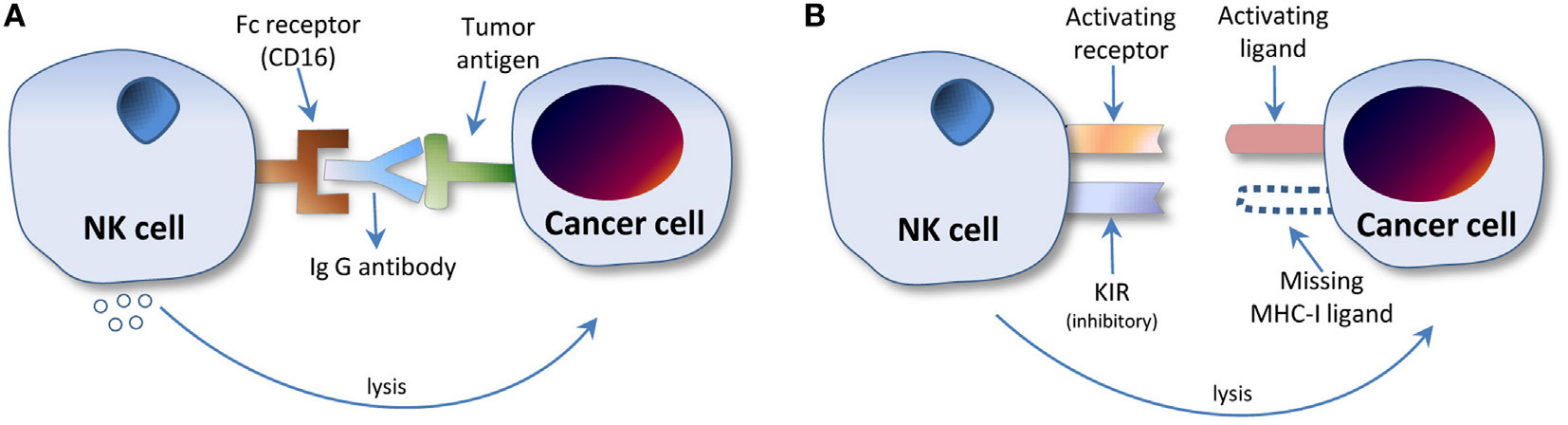
Natural killer (NK) cell interaction with cancer cell microenvironment. **(A)** Antibody-dependent cellular cytotoxicity. The NK cell F_c_ receptor, CD16, binds the F_c_ region of the IgG antibody bound to the tumor antigen, leading to NK cell protease release and subsequent tumor cell lysis. **(B)** NK cell activation *via* natural cytotoxicity receptors (NCRs). The NCR KIR, an inhibitory receptor, recognizes the absence of MHC I ligand on the surface of the cancer cell. Because the inhibitory receptor remains unbound by MHC I, inhibition does not occur, and the NK cell is thus activated, leading to tumor cell lysis. Note: the NK cell killing mechanism proceeds principally by proteolytic lysis. For greater detail on mechanisms of cancer cell killing, see Ref. (9).

Natural killer cells are lymphocyte-like cells considered to be part of the innate immune system, which recognize and respond to abnormal cells—typically either virus-infected or malignant cells—in two ways. First, they bind antibody coated targets *via* immunoglobulin receptors leading to antibody-dependent cellular cytotoxicity (ADCC). Second, they bear natural cytotoxicity receptors (NCRs) that detect the altered expression of ligands on the surface of tumor cells, which ultimately triggers NK cell activation. These mechanisms are illustrated in Figures 1A,B. NCRs are also involved in the process of discriminating between self and non-self *via* the generically termed MHC I receptor and ligand (7, 8).

## Will You Yield and This Avoid: Potentiation of NK Cells

As mentioned above, NK cells are widely circulating lymphoid cells, specialized to eliminate virus-infected and malignant cells. Although traditionally categorized under the umbrella term of the innate immune system, recent evidence has indicated that NK cells can develop prolonged and highly specific memory to various antigens, a property typically associated with the adaptive immune response (10).

Studies have indicated that NK cells are frequently deficient or dysfunctional in patients with malignancy, indicating that this may be a key factor in cancer immunoevasion and progression. In a follow-up study of a patient cohort examining natural cytotoxic activity and cancer incidence, low NK cell function was found to predict an increased risk of developing cancer, thus further supporting their role in malignancy attenuation (11, 12). Given our expanding knowledge of routine NK cell function in conjunction with evidence of consequences of their dysfunction under the circumstances of malignancy, it is reasonable to conclude that the next advancement in cancer therapy should involve NK cell regulation and adaptation. Several approaches for NK cell utilization have been published, and many more are currently being evaluated. Such is the subject for the remainder of this report.

## Go Forth and Multiply!: NK Cell Population Enhancement

For decades, allogeneic hematopoietic cell transplantation (HCT)—the engraftment of a donor’s immune system into a recipient with the objective of eliminating cancer cells—has provided increased disease-free survival to patients with hematologic malignancies. However, such methods come with the risk of graft versus host disease (GVHD), a life-threatening condition wherein recipient cells are recognized as foreign and attacked by the donor immune cells. One potential alternative to this is to isolate specific donor antitumor cells, most notably NK cells, for use in HCT, which minimizes the risk of GVHD. Such methods have displayed positive clinical outcomes in acute myelogenous leukemia (AML) patients who underwent HLA-haploidentical NK cell-specific HCT (11, 13).

Natural killer cells can be obtained from either patients themselves (autologous) or from a donor (allogeneic) and can be derived from multiple sources, including peripheral blood, bone marrow, or umbilical cord blood. Under normal circumstances, few NK cells circulate in human blood, and those that do frequently exhibit limited cytotoxic activity due to immaturity (14). In addition, NK cells are short lived, with an average life span of 2 weeks (15). Thus, research has focused on developing methods to expand NK cell populations, increase their life span, and potentiate their cytotoxicity. Preliminary studies displayed promise with *in vitro* expansion of NK cells using the cytokine IL-2. However, benefit is limited *in vivo* due to the high affinity of T-regulatory cells for IL-2, which is responsible for NK cell inhibition to prevent autoimmunity, as well as by the short half-life—approximately 10 min—of IL-2 in serum (8, 14). Recent evidence has indicated that the inhibition of NK cells can be overcome by diminishing the T-regulatory cell population through the use of an IL-2-diphtheria toxin fusion protein (IL2DT) (16, 17). IL2DT, also known as denileukin difitox or Ontak, is a recombinant cytotoxic fusion protein comprised of IL-2 and diphtheria toxin. The toxin selectively depletes IL-2-receptor expressing cells, particularly those bearing the IL-2 receptor α chain isoform, such as T-regulatory cells. IL2DT has a short half-life, thus donor NK cells infused hours later would be unaffected by it, and in fact, would proliferate more due to the transient decrease in their suppression by T-regulatory cells. A phase II clinical trial from the University of Minnesota that examined 57 patients with refractory AML displayed improvements in rates of NK-cell expansion and AML remission in 27 and 53% of patients, respectively, in those who received IL2DT as compared to 10 and 21%, respectively, in those who did not receive it (16). Other data indicated that modifying NK cells to produce IL-2 prior to transplantation can be done to create a self-sustaining source of IL-2 *in vivo*, providing additional possibilities for combination therapies (8).

Besides solitary IL-2, the combination of the cytokines IL-18, IL-15, and IL-12 has shown promise in inducing the proliferation of memory-like NK cells in immunodeficient mice when accompanied by supplemental exogenous IL-2 (14). Purified human NK cells cultured overnight in IL-2, IL-12, IL-15, and IL-18 that were subsequently infused into mice displayed increased responsiveness to exogenous IL-2; this was thought to be due to the induction of increased CD25 expression on the NK cells, a key component in the formation of the high-affinity IL-2 receptor subtype, IL-2Rαβγ (18). Many other combinations, including both cytokines—most recently IL-27—and other substances such as intravenous immunoglobulin, have been or are currently undergoing evaluation for their prospective roles in NK cell population modulation (14, 19, 20).

## Oh, Sweet Intoxication: Disinhibition of NK Cells

Like true lymphocytes, NK cells have a number of endogenous mechanisms that balance self-defense with self-recognition. First, they express MHC I receptors which interact with different cell types in the surrounding environment. If the MHC I receptors remain unbound upon interaction with a particular cell, that cell is targeted for lysis by the NK cell. Second, they express activating receptors which recognize stress-induced ligands on the surface of target cells and trigger cell lysis upon detection. Finally, they express the activating receptor CD16 that facilitates ADCC upon binding the F_c_ portion of various IgG antibody isotypes. In addition, NK cell activity is also modulated by various cytokines, toll-like receptor ligands, and T-regulatory cells (7, 14). To optimize NK cell antitumor responses, preliminary studies have examined approaches to block NK cell inhibition.

**Figure 2:**
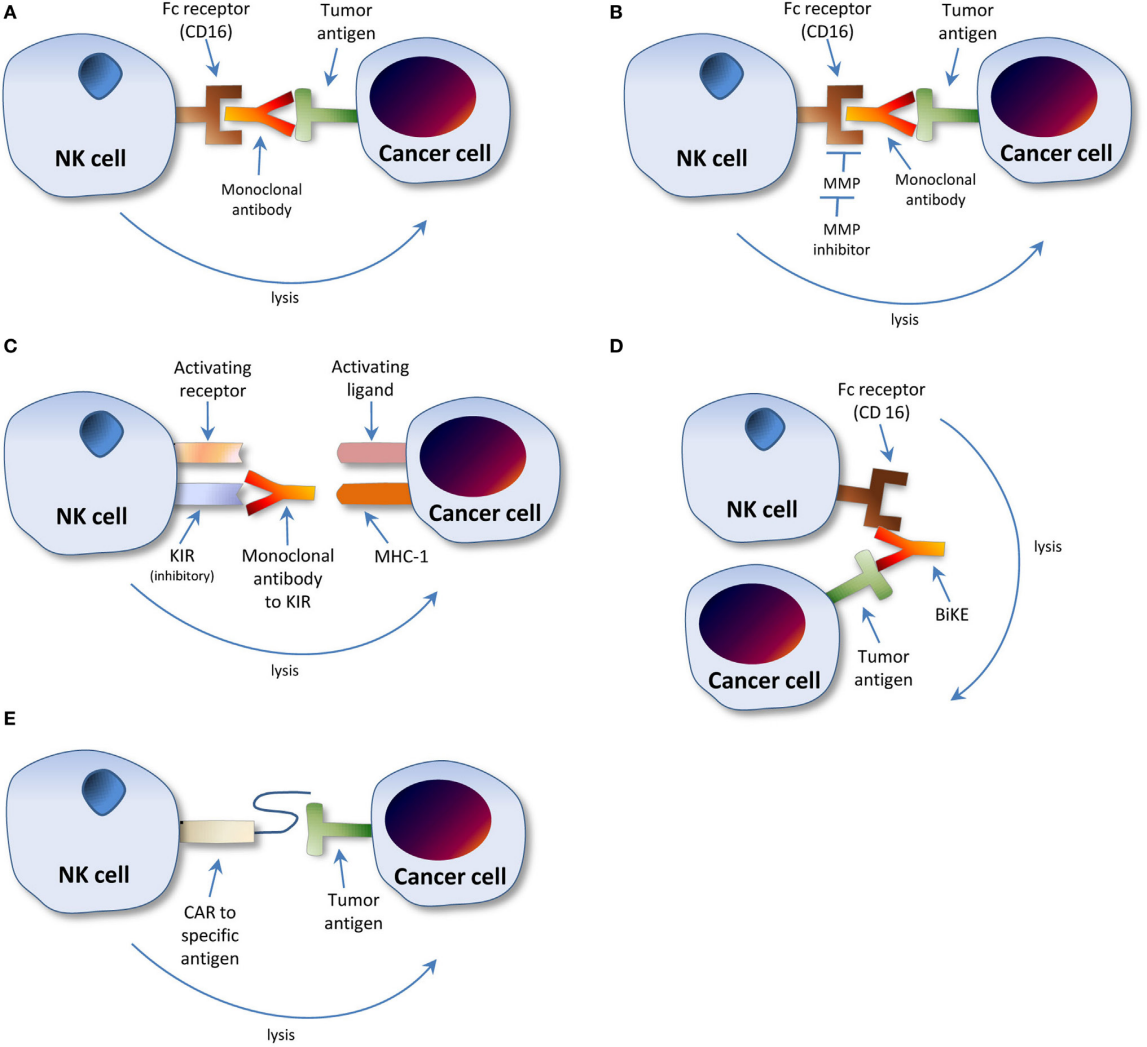
Modulation of natural killer (NK) cell interaction with cancer cell microenvironment. **(A)** Monoclonal antibody (mAb)-driven cellular cytotoxicity. mAbs specific to the tumor antigen bind the tumor cell. The NK cell F_c_ receptor, CD16, binds the F_c_ portion of the mAb bound to the tumor cell, leading to NK cell activation, protease release, and tumor cell lysis. **(B)** Metalloproteinase inhibition combined with mAb therapy. Metalloproteinase inhibitors prevent cleavage and subsequent shedding of CD16 receptors by blocking MMP action. In conjunction with this enhancement in CD16 receptors, mAbs specific to chosen tumor antigen enhance NK cell binding *via* CD16 receptor, leading to NK cell protease release and tumor cell lysis. **(C)** mAb to KIR. The F_ab_ region of an mAb specific to KIR binds the receptor. The antibody blocks KIR from binding the tumor cell MHC I ligand, thus preventing cellular inhibition, consequently leading to NK cell-mediated tumor cell lysis. **(D)** BiKEs and TriKEs. The F_ab_ region of an antibody is designed to target both the F_c_ receptor of the NK cell as well as at least one specific tumor antigen, thus leading to NK cell stimulation and tumor cell lysis. **(E)** Chimeric antigen receptors (CAR). A CAR specific to a chosen tumor antigen is designed and infused into the NK cell, thus enhancing the NK cells’ recognition of the tumor cell. The NK cell then increasingly targets tumor cells for destruction. Note: the NK cell killing mechanism proceeds principally by proteolytic lysis. For greater detail on mechanisms of cancer cell killing, see Ref. (9).

Evidence has shown that the antigen-specific NK cell targeting mechanism *via* CD16 plays a vital role in the effectiveness of established tumor specific monoclonal antibody (mAb) therapies such as trastuzumab, rituximab, and elotuzumab due to CD16 interaction with the F_c_ portion of the mAb that coats the tumor cells, as illustrated in Figure 2A (21). However, several studies have noted the downregulation of CD16 on the NK cells of cancer patients in comparison to healthy patients, making mAb therapies less effective due to decreased ADCC (22, 23). Thus, a developing approach to enhance NK cell-mediated ADCC responsiveness to tumor cells is to inhibit the shedding of CD16 *via* the inhibition of metalloproteinases (MMP) that cleave them, as shown in Figure 2B (21). Furthermore, genotypic variations exist in CD16 between individuals, which can influence its interaction with immunoglobulins; this not only results in variations in mAb therapy effectiveness depending on CD16 genotype but also affords a potential opportunity for targeted therapy (24). Consequently, a multifactorial approach accounting for all of these elements could greatly increase NK cell responses to malignancy.

One such example of these multifactorial strategies in practice is the use of GM6001, an MMP inhibitor used for *in vitro* treatment of NK cells. NK cells treated with GM6001 displayed preserved CD16 expression despite initial losses stimulated by the presence of tumor cells (25). Further studies have confirmed that a specific metalloproteinase, ADAM17, is able to mediate CD16 shedding in NK cells thus providing another promising opportunity for targeted cancer immunotherapy. A preclinical trial that examined the combination of an MMP inhibitor and anti-CTLA4 antibodies in mice with breast cancer displayed delayed tumor growth and metastases reductions in mice treated with MMP inhibitor verses those treated with anti-CTLA4 antibody alone. Anti-CTLA4 antibodies are involved in inhibiting immune system downregulation (26). A phase I/II clinical trial examining an MMP inhibitor which specifically targeted ADAM17 and the similar ADAM10 in combination with trastuzumab in patients with HER2+ metastatic breast cancer displayed no significant improvement from controls (27). While not specific to NK cells, nonetheless these results indicate a necessity for further exploration of this potential therapeutic method.

Another possible, albeit distinctive approach, is the use of an mAb, IPH2101, which blocks KIRs, preventing their binding to MHC I ligands, and thus perpetuating NK cell-induced target cell lysis, as illustrated in Figure 2C. Although initial results using the agent alone were inconclusive, a more recent study combining it with lenalidomide, an anti-angiogenic agent, in patients with multiple myeloma was more promising (11).

Alternative therapies maximizing the activating receptors expressed by NK cells have also been developed. NKG2D is a lectin-like activating receptor expressed by NK cells, activated CD8+ T cells, and macrophages, which reacts to NKG2D ligands found most commonly on tumor cells. Expression of these NKG2D ligands by target cells triggers NK cell cytotoxicity and target cell lysis (28). This mechanism was recently utilized in a study examining the suppression of colon cancer in mice. In the study fluorescent-labeled gene nanoparticles consisting of gene fragments of IL-21, a cytokine involved in lymphocyte activation and tumor suppression, and NKG2D were developed and intravenously injected into mice pretransplanted with tumors. The dsNKG2D-IL-21 nanoparticles preferentially amassed in the tumor cells which could subsequently continue to secrete the dsNKG2D-IL-21 protein, further activating T and NK cells against the tumor tissue. Although further investigation is required, the antitumor effects displayed by such strategies are promising (29).

## Worthy of Recognition: Enhancing Tumor Antigen Recognition

Due to the evidence that NK cells harbor immune memory and antigen recognition properties, therapeutic approaches have now evolved to exploit them. Initial studies utilized KIR mismatch between donors and recipients to enhance donor cell recognition of recipient leukemia, which was found to improve non-relapse mortality and overall survival (11, 30). A prime example of this mechanism in action was shown in an influential study completed by faculty at the University of Perugia and Stanford University, in which 60 high-risk leukemia patients received hematopoietic cell transplants from family donors mismatched by both HLA haplotypes and KIR epitopes. NK cells that were initially shown to lyse allogeneic B-cell lymphoblastoid cells and PHA lymphoblasts in a subset of the patients (4 CML, 4 AML, 5 ALL) were isolated and used to target pretransplant cryopreserved leukemic cells from the same 13 patients. The isolated alloreactive NK cells killed all of the acute and chronic myeloid leukemia samples, and two of five acute lymphoblastic leukemia samples (31).

Knowledge of NK cell triggering *via* CD16 by mAb therapies as discussed above has led to the development of bi- and trispecific antibodies—often referred to as bi- and trispecific specific killer cell engagers (BiKEs and TriKEs)—which enhance the specificity between NK cell and tumor. This method involves designing bi- and trispecific antibodies that fuse the F_ab_ region of the antibody to the specific tumor cell antigen in combination with another F_ab_ region of the same antibody fused with the CD16 portion of NK cells, which leads to NK cell stimulation and tumor cell lysis. Multiple F_ab_ regions can be utilized to specifically target multiple tumor antigens (8). This mechanism is illustrated in Figure 2D. The efficacy of this method was displayed in an *in vitro* study done by the University of Minnesota that combined an MMP inhibitor to ADAM17 with a BiKE targeting CD33 through CD16 in the treatment of refractory AML cells. The combination therapy was found to enhance NK cell cytotoxicity and cytokine release against the CD33+ malignant cells (32).

Another method of enhancing tumor antigen recognition involves the use of single chain fusion proteins—referred to as chimeric antigen receptors (CAR)—molecules artificially engineered and introduced into hematopoietic cells such as NK cells or T lymphocytes to redirect specificity toward a chosen antigen, as shown in Figure 2E (8, 33). CAR modifications of T cells have been researched extensively; however, evaluation of this possibility in NK cells remains in its infancy (8). One recent study which showed much promise involved the transduction of human NK cells with a CAR targeting both wild-type and mutant epidermal growth factor receptors (EGFRs) commonly expressed by glioblastoma (GB) cells. The EGFR-CAR-engineered NK cells displayed increased interferon-γ production and increased tumor cell cytotoxicity when cocultured with GB cells, as well as increased malignant cell growth suppression when administered intracranially in mice (34). Other preclinical studies have successfully transduced human NK cells to express CARs to CD19, CD20, CD244, and HER2, which all displayed efficacy in tumor cell lysis (35). A phase I clinical trial by St. Jude’s in 14 relapsing or refractory B-lymphocyte acute lymphoblastic leukemia patients involving the transduction of NK cells to express a CD19 CAR concluded in February 2015 with no published results to this date (8, 36, 37). A second phase II pilot study also involving the engineering of an anti-CD19 CAR at the National University Health System in Singapore is currently recruiting participants (8, 36, 37).

## Plan for the Future because That is Where You are Going to Spend the Rest of Your Life

As discussed above, mounting evidence indicates that many tumors contain CSCs, rendering them decidedly resistant to established treatments. Recent reports have indicated that CSCs may be preferentially susceptible to NK cell killing, due in part to their decreased expression of MHC class I (3). Furthermore, the stage of cellular differentiation of certain tumor cell types has also been inversely correlated with the degree of NK cell cytotoxicity, with poorly differentiated cells showing significantly higher susceptibility to NK cell-mediated lysis than well-differentiated cells, which appear to display more sensitivity to chemotherapy-mediated cell death (38, 39). Given this evidence, Kozlowska et al. proposed a dual approach combining established chemotherapy/radiotherapy regimens (which better target well-differentiated tumor cells), with immunotherapy (which preferentially eliminates CSCs/poorly differentiated tumor cells) (39). Although these researchers recommended this method with oral cancer specifically in mind, it is not difficult to speculate that widespread use of this methodology may be forthcoming.

## Conclusion

The theory of NK cell immunotherapy has gained momentum in recent years. This review summarized the most prominent methods by which NK cells are being manipulated for potential therapeutic uses, including population propagation, inhibition of NK cell suppression mechanisms, and enhancement of NK cell target recognition. Numerous studies have shown extensive progress in both managing NK cell proliferation and lifespan, as well as in the direct targeting of tumor cells and tissues. Although much has been discovered, there remains much to learn before such therapies will become widespread. Further cultivation of specific NK cell therapies by both biochemical and clinical studies is necessary before dissemination is possible.

In addition, no firm guidelines exist for the application of NK cell-based therapies in practice. While evidence to date seems to support its use as a complementary therapy to existing treatment regimens, the potential for an exclusive NK cell-based polytherapy cannot be ignored.

Finally, more research must be completed on the theory of malignancy itself, and on the individual characteristics of specific tumors. Further evaluation of these unique identifiers may help to determine even more precise therapeutic methods, and form the basis for future tumor staging and treatment guidelines.

## Author Contributions

Both authors have made substantive contributions to the review of literature, which included drafting the manuscript and revising it critically for important intellectual content. Both have given final approval of the version to be published.

## Conflict of Interest Statement

The authors declare that the research was conducted in the absence of any commercial or financial relationships that could be construed as a potential conflict of interest.

## References

[1] Finn OJ. Immuno-oncology: understanding the function and dysfunction of the immune system in cancer. Ann Oncol (2012) 23(Suppl 8):viii6–9. 10.1093/annonc/mds256 22918931 PMC4085883

[2] Mellman I Coukos G Dranoff G Cancer immunotherapy comes of age. Nature (2014) 480(7378):480–9. 10.103/nature10673 PMC3967235 22193102

[3] Luna JI Grossenbacher SK Murphy WJ Canter RJ Targeting cancer stem cells with natural killer cell immunotherapy. Expert Opin Biol Ther (2016) 17:313–24. 10.1080/14712598.2017.1271874 27960589 PMC5311007

[4] Nature Website. Tumor Heterogeneity. (2017). Available from: http://www.nature.com/subjects/tumour-heterogeneity

[5] Shackleton M Quintana E Fearon ER Morrison SJ. Heterogeneity in cancer: cancer stem cells versus clonal evolution. Cell (2009) 138:822–9. 10.1016/j.cell.2009.08.017 19737509

[6] Dick JE Looking ahead in cancer stem cell research. Nat Biotechnol (2009) 27:44–6. 10.1038/nbt0109-44 19131997

[7] Parkin J Cohen B. An overview of the immune system. Lancet (2001) 357:1777–89. 10.1016/S0140-6736(00)04904-7 11403834

[8] Dahlberg CIM Sarhan D Chrobok M Duru AD Alici E. Natural killer cell-based therapies targeting cancer: possible strategies to gain and sustain anti-tumor activity. Front Immunol (2015) 6:605. 10.3389/fimmu.2015.00605 26648934 PMC4663254

[9] Martínez-Lostao L Anel A Pardo J How do cytotoxic lymphocytes kill cancer cells? Clin Cancer Res (2015) 21(22):5048–56. 10.1158/1078-0432.CCR-15-0685 26567364

[10] Paust S von Andrian UH. Natural killer cell memory. Nat Immunol (2011) 12(6):500–8. 10.1038/ni.2032 21739673

[11] Berrien-Elliot MM Romee R Fehniger TA. Improving natural killer cell cancer immunotherapy. Curr Opin Organ Transplant (2015) 20(6):671–80. 10.1097/MOT.0000000000000243 26414502 PMC4635041

[12] Imai K Matsuyama S Miyake S Suga K Nakachi K Natural cytotoxic activity of peripheral-blood lymphocytes and cancer incidence: an 11-year follow-up study of a general population. Lancet (2000) 356(9244):1795–9. 10.1016/S0140-6736(00)03231-1 11117911

[13] Rubnitz JE Inaba H Ribeiro RC Pounds S Rooney B Bell T NKAML: a pilot study to determine the safety and feasibility of haploidentical natural killer cell transplantation in childhood acute myeloid leukemia. J Clin Oncol (2010) 28:955–9. 10.1200/JCO.2009.24.4590 20085940 PMC2834435

[14] Guillerey C Huntington ND Smyth MJ. Targeting natural killer cells in cancer immunotherapy. Nat Immunol (2016) 17(9):1025–36. 10.1038/ni.3518 27540992

[15] Vogel B Tennert K Full F Ensser A Efficient generation of human natural killer cell lines by viral transformation. Leukemia (2014) 28:192–5. 10.1038/leu.2013.188 23787393

[16] Bachanova V Cooley S Defor TE Verneris MR Zhang B McKenna DH Clearance of acute myeloid leukemia by haploidentical natural killer cells is improved using IL-2 diphtheria toxin fusion protein. Blood (2014) 123(25):3855–63. 10.1182/blood-2013-10-532531 24719405 PMC4064329

[17] Wei M Marino J Trowell A Zhang H Peraino JS Rajasekera PV Diphtheria toxin-based recombinant murine IL-2 fusion toxin for depleting murine regulatory T cells in vivo. Protein Eng Des Sel (2014) 27(9):289–95. 10.1093/protein/gzu034 25147093

[18] Leong JW Chase JM Romee R Schneider SE Sullivan RP Cooper MA Preactivation with IL-12, IL-15, and IL-18 induces CD25 and a functional high-affinity IL-2 receptor on human cytokine-induced memory-like natural killer cells. Biol Blood Marrow Transplant (2014) 20(4):463–73. 10.1016/j.bbmt.2014.01.006 24434782 PMC3959288

[19] Zwirner NW Ziblat A Regulation of NK cell activation and effector functions by the IL-12 family of cytokines: the case of IL-27. Front Immunol (2017) 8:25 10.3389/fimmu.2017.00025 28154569 PMC5243847

[20] Issekutz A Derfalvi B Käsermann F Rowter D Potentiation of cytokine-induced proliferation of human natural killer cells by intravenous immunoglobulin G. Clin Immunol (2015) 161(2):373–83. 10.1016/j.clim.2015.08.005 26307433

[21] James AM Cohen AD Campbell KS. Combination immune therapies to enhance anti-tumor responses by NK cells. Front Immunol (2013) 4:481. 10.3389/fimmu.2013.00481 24391651 PMC3870292

[22] Watanabe M Kono K Kawaguchi Y Mizukami Y Mimura K Maruyama T NK cell dysfunction with down-regulated CD16 and up-regulated CD56 molecules in patients with esophageal squamous cell carcinoma. Dis Esophagus (2010) 23(8):675–81. 10.1111/j.1442-2050.2010.01073.x 20545975

[23] Petricevic B Laengle J Singer J Sachet M Fazekas J Steger G Trastuzumab mediates antibody-dependent cell-mediated cytotoxicity and phagocytosis to the same extent in both adjuvant and metastatic HER2/neu breast cancer patients. J Transl Med (2013) 11:307. 10.1186/1479-5876-11-307 24330813 PMC4029549

[24] Wang W Erbe AK Hank JA Morris ZS Sondel PM NK cell-mediated antibody-dependent cellular cytotoxicity in cancer immunotherapy. Front Immunol (2015) 6:368 10.3389/fimmu.2015.00368 26284063 PMC4515552

[25] Zhou Q Gil-Krzewska A Peruzzi G Borrego F. Matrix metalloproteinases inhibition promotes the polyfunctionality of human natural killer cells in therapeutic antibody-based anti-tumour immunotherapy. Clin Exp Immunol (2013) 173(1):131–9. 10.1111/cei.12095 23607800 PMC3694543

[26] Li M Xing S Zhang H Shang S Li X Ren B A matrix metalloproteinase inhibitor enhances anti-cytotoxic T lymphocyte antigen-4 antibody immunotherapy in breast cancer by reprogramming the tumor microenvironment. Oncol Rep (2016) 35(3):1329–39. 10.3892/or.2016.4547 26752000 PMC4750755

[27] Miller MA Sullivan RJ Lauffenburger DA Molecular pathways: receptor ectodomain shedding in treatment, resistance, and monitoring of cancer. Clin Cancer Res (2017) 23(3). 10.1158/1078-0432.CCR-16-0869 PMC5290119 27895032

[28] Diefenbach A Jamieson AM Liu SD Shastri N Raulet DH. Ligands for the murine NKG2D receptor: expression by tumor cells and activation of NK cells and macrophages. Nat Immunol (2000) 1(2):119–26. 10.1038/77793 11248803

[29] Tan L Han S Ding S Xiao W Ding Y Qian L Chitosan nanoparticle-based delivery of fused NKG2D-IL-21 gene suppresses colon cancer growth in mice. Int J Nanomedicine (2017) 12:3095–107. 10.2147/IJN.S128032 28450784 PMC5399983

[30] Mancusi A Ruggeri L Urbani E Pierini A Massei MS Carotti A Haploidentical hematopoietic transplantation from KIR ligand-mismatched donors with activating KIRs reduces nonrelapse mortality. Blood (2015) 125:3173–82. 10.1182/blood-2014-09-599993 25769621

[31] Ruggeri L Capanni M Casucci M Volpi I Tosti A Perruccio K Role of natural killer alloreactivity in HLA-mismatched hematopoietic stem cell transplantation. Blood (1999) 94(1):333–9. 10381530

[32] Wiernek A Foley B Zhang B Verneris MR Warlick E Gleason MK targeting natural killer cells to acute myeloid leukemia *in vitro* with a CD16x33 bispecific killer cell engager (BiKE) and ADAM17 inhibition. Clin Cancer Res (2013) 19(14):3844–55. 10.1158/1078-0432.CCR-13-0505 23690482 PMC3715574

[33] Cooper LJN Maiti SN Chimeric antigen receptors on T cells. In: Schwab M, editor. Encyclopedia of Cancer. 3rd ed. Berlin, Germany: Springer Berlin Heidelberg (2011). p. 806–10.

[34] Han J Chu J Chan WK Zhang J Wang Y Cohen JB CAR-engineered NK cells targeting wild-type EGFR and EGFRvIII enhance killing of glioblastoma and patient-derived glioblastoma stem cells. Sci Rep (2015) 5:11483. 10.1038/srep11483 26155832 PMC4496728

[35] Rezvani K Rouce RH. The application of natural killer cell immunotherapy for the treatment of cancer. Front Immunol (2015) 6:578. 10.3389/fimmu.2015.00578 26635792 PMC4648067

[36] U.S. National Institutes of Health. Genetically Modified Haploidentical Natural Killer Cell Infusions for B-Lineage Acute Lymphoblastic Leukemia. ClinicalTrials.gov (2017). Available from: https://clinicaltrials.gov/ct2/show/NCT00995137.

[37] U.S. National Institutes of Health. Pilot Study of Redirected Haploidentical Natural Killer Cell Infusions for B-Cell Lineage Acute Lymphoblastic Leukemia. ClinicalTrials.gov (2016). Available form: https://clinicaltrials.gov/ct2/show/NCT01974479.

[38] Jewett A Man Y Cacalano N Kos J Tseng H. Natural killer cells as effectors of selection and differentiation of stem cells: role in resolution of inflammation. J Immunotoxicol (2014) 11(4):297–307. 10.3109/1547691X.2013.877104 24575813

[39] Kozlowska AK Topchyan P Kaur K Tseng HC Teruel A Hiraga T Differentiation by NK cells is a prerequisite for effective targeting of cancer stem cells/poorly differentiated tumors by chemopreventive and chemotherapeutic drugs. J Cancer (2017) 8(4):537–54. 10.7150/jca.15989 28367234 PMC5370498

